# Supplementary material for: Multi-Targeting Neuroprotective Effects of Syzygium aromaticum Bud Extracts and Their Key Phytocompounds against Neurodegenerative Diseases
Source: Int J Mol Sci. 2023 May 2;24(9):8148. doi: 10.3390/ijms24098148 (PMC10178913; doi:10.3390/ijms24098148)
Supplement: Supplementary file 1 [file ijms-24-08148-s001.zip › ijms-2357672-supplementary.pdf]

Supplementary information

Supplementary Figure S1. Phytoconstituents identified in (A) CL-EA and (B) CL-H extract from *Syzygium aromaticum* buds using gas chromatography-mass spectrometry.

A

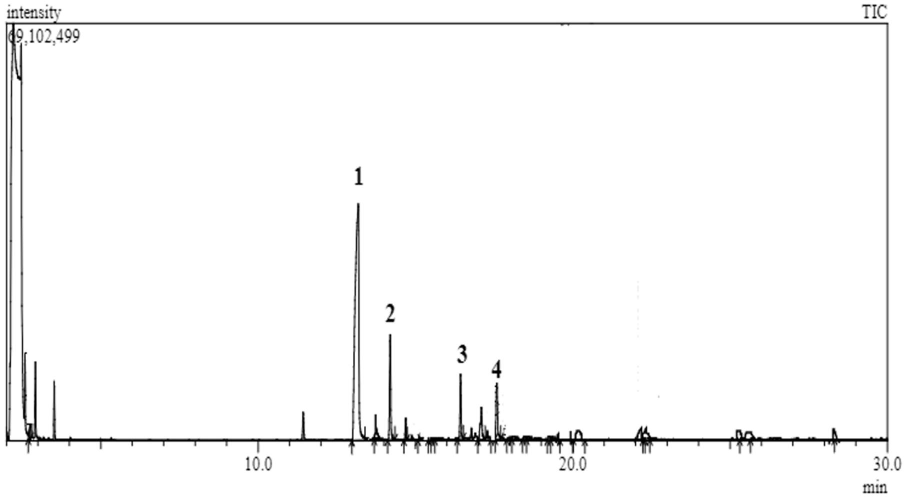

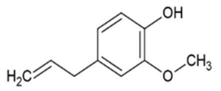

1

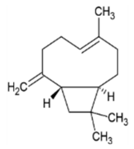

2

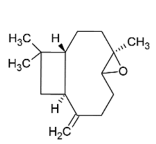

3

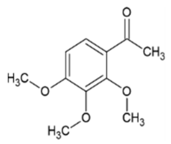

4

Peak Report TIC						
**Major Peak no.**	**R.Time**	**Area**	**Area (%)**	**Height**	**Height (%)**	**Name**
	2.743	6112358	1.16	2316736	2.01	Oxalic acid, monoamide, n-propyl, pentyl ester
**1**	**13.185**	**311542886**	**59.32**	**39130217**	**33.98**	**Eugenol**
	13.725	1988913	0.38	870766	0.76	Vanillin
**2**	**14.2**	**50249612**	**9.57**	**18563146**	**16.12**	**Caryophyllene**
	14.694	8004729	1.52	3601283	3.13	Humulene
	15.128	1160086	0.22	458020	0.4	.alpha.-Farnesene

	15.451	6657888	1.27	2252939	1.96	.alfa.-Copaene
	15.529	3040768	0.58	1183519	1.03	cis-Calamenene
	15.676	3114138	0.59	1403529	1.22	Cubenene
**3**	**16.435**	**27721704**	**5.28**	**10998928**	**9.55**	**Caryophyllene oxide**
	17.097	20730339	3.95	5756851	5	Caryophylla-4(12),8(13)-dien-5.alpha.-ol
	17.46	10436910	1.99	3855739	3.35	(-)-Globulol
**4**	**17.589**	**31889618**	**6.07**	**10276552**	**8.92**	**2',3',4' Trimethoxyacetophenone**
	17.961	1581228	0.3	308970	0.27	Ethyl homovanillate
	18.125	3835516	0.73	1368803	1.19	(E)-4-(3-Hydroxyprop-1-en-1-yl)-2-methoxyphenol
	18.468	3168387	0.6	1126725	0.98	Deoxyqinghaosu
	18.587	2415122	0.46	828828	0.72	Ledene oxide-(II)
	19.253	4308143	0.82	1754673	1.52	2,6,10,15,19,23-Hexamethyl-tetracosa-2,10,14,18,22-pentaene-6,7-diol
	19.338	1037695	0.2	366649	0.32	Epiglobulol
	19.635	1446073	0.28	618103	0.54	1-(4-Acetoxy-3-methoxyphenyl)allyl acetate
	20.068	1252840	0.24	532972	0.46	(3S,3aS,6R,7R,9aS)-1,1,7-Trimethyldecahydro-3a,7-methanocyclopenta[8]annulene-3,6-diol
	20.492	7138554	1.36	2309704	2.01	Pentadecanoic acid
	22.272	3088742	0.59	1276908	1.11	Linoelaidic acid
	22.33	5507793	1.05	1492674	1.3	cis-9-Hexadecenal
	22.529	2298071	0.44	635463	0.55	Z,Z-8,10-Hexadecadien-1-ol
	25.371	1787959	0.34	690363	0.6	Benzonitrile, m-phenethyl-
	25.687	1119045	0.21	407691	0.35	Anethole
	28.397	2511706	0.48	759977	0.66	3-Allyl-6-methoxyphenol
		525146823	100	115146728	100	

B

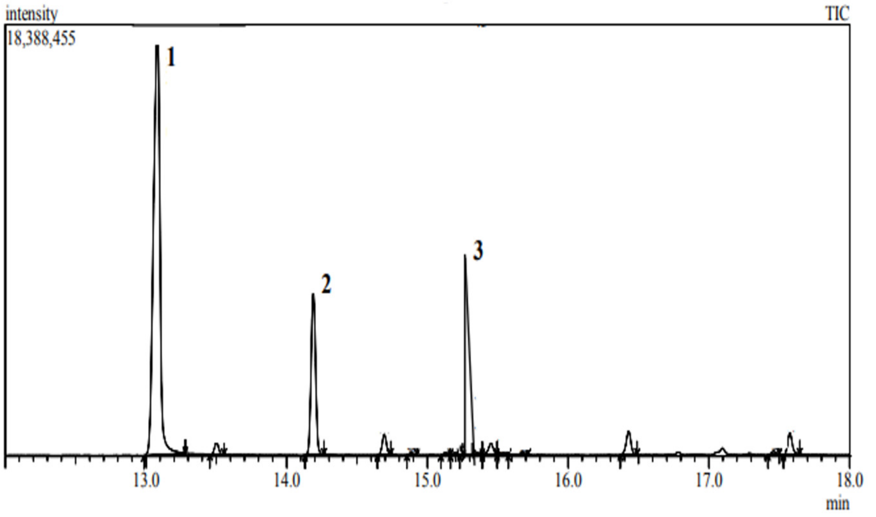

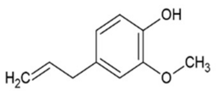

1

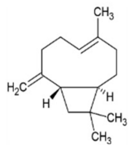

2

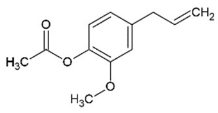

3

Peak Report TIC						
**Major Peak no.**	**R.Time**	**Area**	**Area (%)**	**Height**	**Height (%)**	**Name**
**1**	**13.082**	**57686726**	**57.46**	**18322133**	**48.93**	**Eugenol**
	13.502	1050351	1.05	520186	1.39	.alfa.-Copaene
**2**	**14.189**	**15301912**	**15.24**	**6996474**	**18.69**	**Caryophyllene**
	14.693	1805864	1.8	874229	2.33	Humulene
	14.887	176576	0.18	95409	0.25	Copaene
	15.13	239854	0.24	107870	0.29	.alpha.-Farnesene
	15.197	238987	0.24	104299	0.28	(3R,3aR,3bR,4S,7R,7aR)-4-Isopropyl-3,7-dimethyloctahydro-1H-cyclopenta[1,3]cyclopropa[1,2]benzen-3-ol
**3**	**15.288**	**17269865**	**17.2**	**7536787**	**20.13**	**Eugenyl acetate**
	15.452	1265188	1.26	492394	1.32	1-Isopropyl-4,7-dimethyl-1,2,3,5,6,8a-hexahydronaphthalene
	15.528	377278	0.38	164696	0.44	cis-Calamenene
	15.676	234185	0.23	128659	0.34	Cubenene
	16.43	2358271	2.35	1021698	2.73	Caryophyllene oxide
	17.459	333055	0.33	155747	0.42	(-)-Globulol
	17.575	2049815	2.04	922087	2.46	2',3',4' Trimethoxyacetophenone
		100387927	100	37442668	100	
